# Tripal and Galaxy: supporting reproducible scientific workflows for community biological databases

**DOI:** 10.1093/database/baaa032

**Published:** 2020-07-04

**Authors:** Shawna Spoor, Connor Wytko, Brian Soto, Ming Chen, Abdullah Almsaeed, Bradford Condon, Nic Herndon, Heidi Hough, Sook Jung, Meg Staton, Jill Wegrzyn, Dorrie Main, F Alex Feltus, Stephen P Ficklin

**Affiliations:** 1 Dept of Horticulture, Washington State University, 149 Johnson Hall 646414, Pullman, WA 99164-6414, USA; 2 Entomology and Plant Pathology, University of Tennessee, 2505, 370 E J. Chapman Dr Plant Biotechnology Building, Knoxville, TN 37996, USA; 3 Ecology and Evolutionary Biology, University of Connecticut, 75 N. Eagleville Road, Unit 3043 Storrs, CT 06269-3043, USA; 4 Dept of Genetics and Biochemistry, Clemson University, 154 Poole Agricultural Center Clemson, SC 29634, USA; 5 Dept of Computer Science, East Carolina University, College of Engineering and Technology East 5th Street Greenville, NC 27858-4353, USA

## Abstract

Online biological databases housing genomics, genetic and breeding data can be constructed using the Tripal toolkit. Tripal is an open-source, internationally developed framework that implements FAIR data principles and is meant to ease the burden of constructing such websites for research communities. Use of a common, open framework improves the sustainability and manageability of such as site. Site developers can create extensions for their site and in turn share those extensions with others. One challenge that community databases often face is the need to provide tools for their users that analyze increasingly larger datasets using multiple software tools strung together in a scientific workflow on complicated computational resources. The Tripal Galaxy module, a ‘plug-in’ for Tripal, meets this need through integration of Tripal with the Galaxy Project workflow management system. Site developers can create workflows appropriate to the needs of their community using Galaxy and then share those for execution on their Tripal sites via automatically constructed, but configurable, web forms or using an application programming interface to power web-based analytical applications. The Tripal Galaxy module helps reduce duplication of effort by allowing site developers to spend time constructing workflows and building their applications rather than rebuilding infrastructure for job management of multi-step applications.

## Introduction

The execution of scientific workflows is increasingly important in biological research, especially as the costs for data acquisition are decreasing and the quantity and complexity of data is increasing. The fields of genomics, genetics and phenomics are important generators of large-scale data in biology as evidenced, in genomics, by more than 31 petabytes of DNA sequence deposited into the NCBI Sequence Read Archive (SRA) (1) archive in the last decade. These data often require multiple software tools for preprocessing and analysis as data are transformed as they move from one tool to the other. Management of data, in a manual fashion, as it progresses from one tool to the next, in a reproducible manner, can be cumbersome and fraught with human error. Scientists are generally able to access, combine and move data as needed, yet the Findabilty, Accessibility, Interoperability and Reproducibility (FAIR) (2) of results is inconsistent.

Additionally, scientists have varying access to computational resources which may include stand-alone workstations, institutional HPC clusters, national-level infrastructure such as XSEDE (3), JetStream (4), the Open Science Grid (5) and The Pacific Research Platform (6) (as in the USA) or to commercial cloud computing providers. Navigating these different infrastructures to execute scientific workflows can be time-consuming for life scientists, who may not have the training nor resources to move their workflows from one environment to another.

To help ease the burden for scientists, software tools are increasingly available to support data management, computing management and the FAIR-ness of scientific workflows. Some of these include Kepler (7), SnakeMake (8), Nextflow (9), the Galaxy Project (10), Pegasus (11) and others. Of these, Galaxy has proven to be popular among biology researchers primary because of its intuitive, easy-to-use web-based interface that abstracts access to UNIX and its high-performance computing back-end. Galaxy can be installed by anyone and supports execution of workflows on stand-alone workstations, HPC systems, and the cloud. Typically, researchers access a Galaxy instance through a provider such as an installation at their local institution or a public server such as the Use Galaxy service (https://usegalaxy.org/), powered by the Texas Advanced Computing Center (TACC) and CyVerse (12)—they need not install their own instance. Galaxy supports FAIR data by allowing researchers to publish results and share their workflows.

For complex workflows, datasets are often sourced from multiple locations that include online data repositories, in-house generated datasets or data shared by collaborators. For example, for Differential Gene Expression (DGE) analysis (13), genomic or transcriptomic reference datasets are often retrieved from online genomic databases, while the gene expression data are generated through in-house experiments or retrieved from other repositories such as the NCBI Sequence Read Archive (SRA). Galaxy provides a mechanism by which online databases can share data for integration with a workflow in Galaxy. This is extremely beneficial for scientists as they need not leave the Galaxy platform, in such a case, in order to find data.

Despite the benefits of Galaxy and other scientific workflow tools, there is a need for the online data repository (or database) to provide analytical tools for their users. When online genome databases first appeared in the 1990s, they often included computational tools that directly used the data they provided. The most common tool offered was an online BLAST (14, 15) tool that was preloaded with data from the repository. Additionally, genome databases often provided a variety of other tools which consisted of popular software that were ‘wrapped’ for presentation on the web by in-house programmers. As data sizes have increased, the complexity of wrapping tools for the web becomes more difficult. As online databases search for better approaches for sustainability, any duplication of effort for wrapping the same workflows is inefficient.

To this end, the Tripal (16–18) platform provides a mechanism by which online genomic, genetic and breeding databases can reduce duplication of effort, develop a common platform and improve the sustainability of their respective online databases. It is a software platform, developed using PHP, the Drupal content management system (CMS) and the Chado database schema (19). It is meant to be installed by anyone who wishes to provide an online data repository for a research community and strives to meet FAIR data standards. An international group of developers collaborate in an officially organized community to develop the Tripal core package and a variety of extension modules. Tripal currently supports databases around the world housing genomic, genetic and breeding data for hundreds of species. Some examples of Tripal sites include Banana Genome Hub (20), Cucurbit Genomics (21), the Genome Database for Rosaceae (22–24), Hardwood Genomics (25), i5k Workspace (26), KnowPulse (27), Plansosphere (28), the Rice Genome Hub (29), TreeGenes (30) and many others. As Tripal is used by multiple data repositories, it provides a unique opportunity for Tripal site developers to share infrastructure with other Tripal sites. This sharing is significant for sites that house data and provide services for orphaned or poorly funded research communities.

As with all online data repositories, Tripal-based sites may want to provide analytical tools to their users, yet due to the complexity of modern large-data workflows, this becomes challenging, especially for the smaller teams that typically manage a Tripal-based site. To this end, the Tripal Galaxy extension module was created to support ‘wrapping’ of workflows that are available in a remote Galaxy instance. Tripal-based sites can integrate with Galaxy workflows in two different ways: first, via auto-generated web forms for commonly executed workflows, education and training or clinical applications, and second via the Tripal Galaxy API to power online web applications. Here we describe these two approaches with brief usage examples. The Tripal Galaxy module source code is available on GitHub at https://github.com/tripal/tripal_galaxy with full online documentation at https://tripal-galaxy.readthedocs.io/en/latest/. It is a Gold rated module (the highest level) according to the current Tripal Module Rating System.

## Usage

### Implementation summary

The Tripal Galaxy module is an extension module for Tripal. Thus, any Tripal-based site can download, install and enable the module and quickly add support for Galaxy workflows with minimal effort. The Tripal Galaxy module uses the Blend4php library (31), also written by some of the co-authors of this manuscript, which is a library of PHP wrapper functions for the Galaxy application programming interface (API). It is modeled after BioBlend (32, 33) which provides similar wrappers for the Java language. The Tripal Galaxy module can automatically create web forms for any workflows already present in a Galaxy instance and provides an API that allows a site developer to use Galaxy workflows to power an online application. The module uses the Tripal file quota system to prevent filling of available storage space, supports sharing of site-specific files for workflows and sets time limits to clean up and expire old workflow executions. Administrative reports provide an overview of popular workflows and usage statistics.

### Auto web form creation

The first method for integration with Galaxy is through automatic generation of web forms for a specific Galaxy workflow. With this method, a Tripal-site developer can specify an existing workflow on a remote Galaxy server and the module will automatically create a step-by-step web form that an end user can use to upload or specify input data, set parameters and launch the workflow. There are several reasons why a site developer may want to provide a web form within their site rather than redirecting the user to a Galaxy instance. First, using a web form within the site’s visual theme maintains the end user experience. Second, the site developer can control the workflows the site is willing to support. This can be helpful for sites that receive data from their users for inclusion in the site. By ensuring that workflows follow proper community-accepted standards and protocols, the site can more easily verify the quality of input data and reduce the time required by site curators. Results that already reside on the site are more easily integrated. Third, by providing commonly used workflows, the site can easily provide site-specific data for use in workflows for their users, thus easing the data management and computational burden that some of their users may have difficulties with. Fourth, workflows can be used for educational purposes. Site visitors can be trained in analytical concepts without the burden of learning Galaxy or HPC systems. Fifth, web form workflows might be more appropriate for a clinical setting, where clinicians need not be distracted by the wealth of functionality in Galaxy.

**Figure 1:**
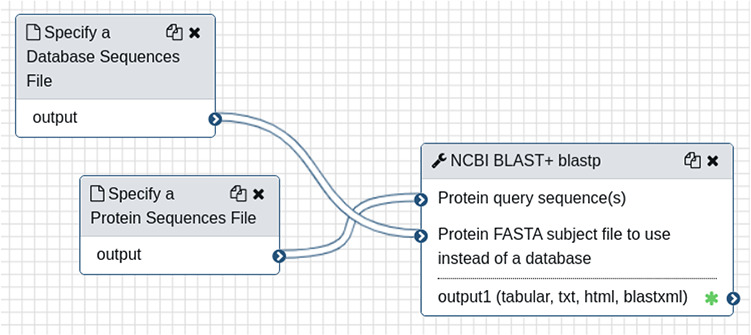
A simple workflow, as shown in the Galaxy online interface, consisting only of the NCBI BLAST+ tool. It requires two input files: a query protein sequence and protein database—both in FASTA format.

**Figure 2:**
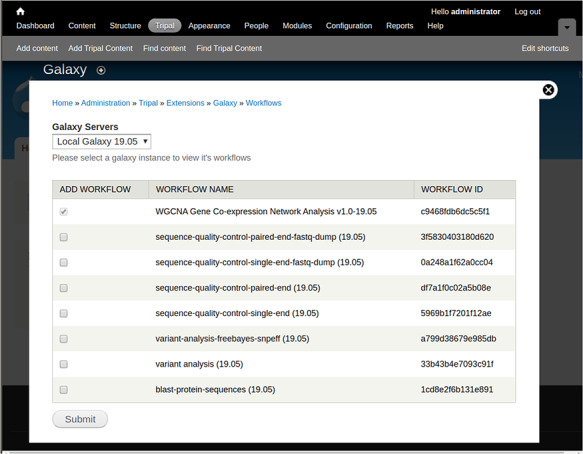
A screenshot of the interface that allows a site developer to select workflows for integration with Tripal.

To demonstrate creation and use of web forms, a small Galaxy-created NCBI BLAST workflow is shown in Figure 1. The workflow requires two input files, a protein query and protein database, followed by the blastp tool. To integrate this workflow for automatic web form creation, the site developer would first provide connection details using a web interface provided by the Tripal Galaxy module, including the URL, username and API Key. It is recommended that the Galaxy account be specific to the Tripal site and not tied to a specific person in order to keep results in the histories for Tripal-submitted workflows separate from any individual account. Next, the site developer can use the module’s web interface to connect to the remote Galaxy server and select the workflow to be integrated. Figure 2 provides an example of several workflows available using the provided credentials on a locally installed Galaxy service, including the BLAST workflow shown in Figure 1.

**Figure 3:**
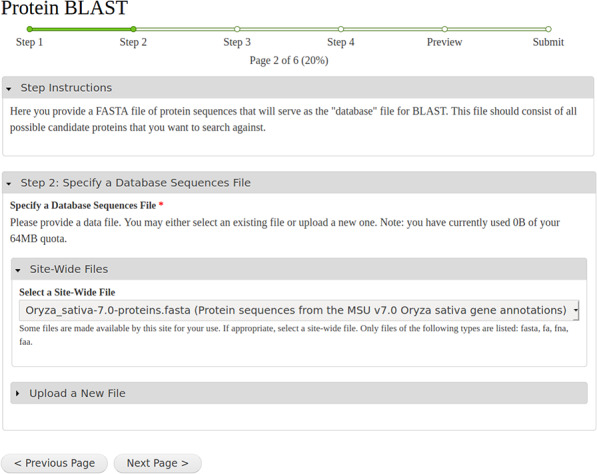
A screenshot of the step-by-step web form created for the NCBI BLAST example workflow shown in Figure 1.

After a workflow is selected, a step-by-step web form is created. As an example, Figure 3 shows a screenshot of the second step of the BLAST workflow, which provides a tool for selection of the protein database input file. A progress bar, listing several steps, is visible near the top of the form. The first step in any workflow is always instructions that the site developer can customize for their site’s end users. In the BLAST example, Steps 2 and 3 are for specifying and uploading the database and query protein sequences. Figure 3 shows expandable field sets that allow a user to upload a new file, use already provided site-wide files or use an existing file they have uploaded in the past. Step 4 is for customizing the BLAST settings. The last step is a Preview step showing all selected settings. For multi-tool workflows, this preview becomes especially important as it provides an overview of all the parameters set for all tools in the web form. Once a user reviews the settings in the Preview step, they can submit the workflow for execution.

**Figure 4:**
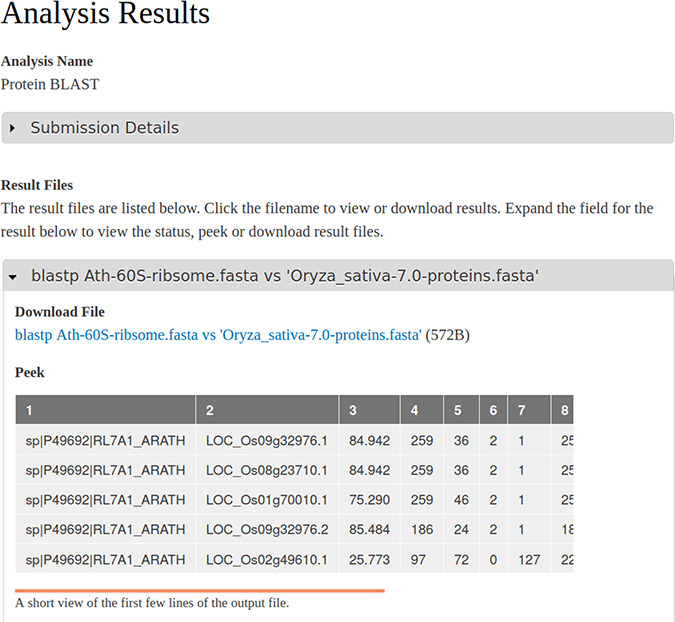
A screenshot of the results page resulting from an execution of the BLAST workflow that is shown in Figure 1 and submitted via the Tripal Galaxy module by an end user. The table in the ‘peek’ section is provided by the Galaxy server.

When a workflow begins or terminates (either successful or failure) the user receives an email informing of the status. Site administrators can customize the text of these emails for each workflow, if desired, using the Tripal Galaxy administrative interface. During execution, Galaxy houses results within a storage concept referred to as a ‘history’, and each invocation of a workflow via Tripal uses a unique history. Therefore, results for all workflows are kept separate from one another, and the entire set of files (both intermediate and final) for a single workflow execution are available to the end user. Upon successful completion, the user can view the status of their workflow from their site profile pages and click a link to view the results page. If an error occurs, the end user (and site administrator) can view the error on the results page. The Tripal Galaxy module will provide all final and intermediate result files that are present in the workflow history. Figure 4 shows a screenshot of results from the example BLAST workflow.

**Table 1:** The list of API functions for managing workflows on a remote Galaxy instance

**Function name**	**Purpose**
**Galaxy server and workflow setup**	
tripal_galaxy_add_galaxy	Adds a remote Galaxy server.
tripal_galaxy_get_galaxies	Retrieves the list of integrated Galaxy servers.
tripal_galaxy_get_connection	Connect to a remote Galaxy server.
tripal_galaxy_test_connection	Tests if a Galaxy server is still active.
tripal_galaxy_add_workflow	Adds a remote Galaxy workflow to Tripal.
**Workflow submission**	
tripal_galaxy_init_submission	Initializes a workflow for execution.
tripal_galaxy_create_history	Used to create a history for workflow results.
tripal_galaxy_upload_file	Uploads files for a workflow to a Galaxy server.
tripal_galaxy_get_workflow_defaults	Retrieves an array for a workflow populated with defaults.
tripal_galaxy_invoke_workflow	Submits a workflow for execution.
**Status and results**	
tripal_galaxy_check_submission_status	Check the progress of a workflow on the Galaxy server.
tripal_galaxy_get_datasets	Retrieves a list of details about result files.
tripal_galaxy_download_file	Used to retrieve a single result file.
tripal_galaxy_get_proxy_url	Provides a URL for download of files via the web interface.

**Table 2:** Available resources for the Tripal Galaxy module

Resource name	Location
Tripal Galaxy user’s documentation	https://tripal-galaxy.readthedocs.io/
Tripal Galaxy source code repository	https://github.com/tripal/tripal_galaxy
Galaxy workflows used on HWG.org	https://github.com/statonlab/galaxy-workflows
Aurora Tools source code repository	https://github.com/statonlab/aurora-galaxy-tools
Aurora Tools demonstration page	https://statonlab.github.io/aurora-galaxy-tools/
Blend4php API documentation	http://galaxyproject.github.io/blend4php/docs-v0.1a/html/index.html
Blend4php source code repository	https://github.com/galaxyproject/blend4php
Tripal User’s Guide	https://tripal.readthedocs.io/
Tripal Developer Handbook	https://tripal.readthedocs.io/en/latest/dev_guide.html
Contributing to the Tripal community	https://tripal.readthedocs.io/en/latest/contributing.html
The Galaxy Project home portal	https://galaxyproject.org/
UseGalaxy.org public Galaxy service	https://usegalaxy.org/

Unfortunately, the end results of a workflow are not always best understood via a list of files that the end user must peruse. Often, summary statistics, coupled with results, housed in a descriptive report will best serve the end user. The Tripal Galaxy module does not provide any visualization or summarization of results into reports. For this purpose, the Aurora Galaxy Tools toolkit (34) exists. The Aurora Tools was created by some of the co-authors of the manuscript and enables a Galaxy workflow developer, who is aware that results will be viewed from within a Tripal site, to construct reports using an R Markdown framework. The Tripal Galaxy module can display these reports from within the results page. Aurora Tools reports are also visible from within Galaxy and can be useful for workflow developers outside of the Tripal community as well.

### Powering an application

In some cases, a Tripal-based site developer may wish to create a PHP-based web application that is meant to use a scientific workflow ‘behind-the-scenes’. The end user, in this case, does not interact with a web form as previously shown, but rather interacts with the web application. The web application integrates with the Tripal site and allows the end user to execute tasks through some custom interface. Those tasks are then executed on behalf of the user using pre-built Galaxy workflows that are also created by the site developer. The web application can communicate with the Galaxy server using the Tripal Galaxy API function set—a set of PHP functions. The API enables the programmer to upload the necessary files, initialize the workflow with appropriate parameters, launch the workflow, check its status and retrieve results once completed. The programmer can therefore customize the experience the web application provides to the user without exposing the Galaxy or Tripal Galaxy interfaces. Table 1 provides an overview of the API functions and their role. The Tripal Galaxy online documentation provides two major subsections to help programmers use the API. First is a reference that describes each function, including function arguments, and return values. Second, is a tutorial providing step-by-step code examples for each of the functions. These resources are found in the Tripal Galaxy online documentation at https://tripal-galaxy.readthedocs.io/en/latest/API/api.html.

The Tripal Galaxy API is currently in use for the CartograTree application (https://treegenesdb.org/ct) (35, 36) on the TreeGenes website. CarograTree is an online tool that integrates phenotype, genotype and environmental data for georeferenced trees to assess population structure, association mapping and environmental adaptation. CartograTree provides an interactive map of the world, with optional GIS layers and tree populations associated with published population-level investigations. The selected datasets can be evaluated via their metadata and directed to the appropriate workflows.

### Administrative features

For either approach, the Tripal Galaxy module provides a variety of administrative features. First, as previously mentioned, site administrators can add remote Galaxy servers such that Tripal knows about them. This in turn makes remote workflows discoverable for integration on the site. Galaxy servers and workflows that are added via the Tripal Galaxy API will also be visible via the administrative pages.

Second, a job queue is available that provides to site administrators a listing of active, completed, and deleted workflows. This will include jobs added by users via the web form or by web applications using the API. From this job queue, the site administrator can view results from any executed workflow, view error logs (if necessary) and view the parameter settings for each submission.

Third, a report page is provided that includes usage graphs of the top workflow submitters (users) on the site, and the top workflows submitted. These reports will include both web form submitted workflows and those submitted by the API. They can help site administrators gauge usage and popularity of workflows.

Fourth, a form is provided to specify site-specific files that should be made available to all users. Site-specific files will appear in the data steps of each workflow as shown in Figure 2. This can prevent end users from uploading the same files repeatedly or files that are already present on the site.

Finally, the site administrator can specify the number of days a history will remain on a remote Galaxy server. Once the set number of days has passed, the Tripal Galaxy module will automatically delete the history and results will no longer be available for viewing or download. This helps ensure that storage space on the remote Galaxy server does not fill with older workflow results. Similarly, Tripal itself provides a quota system for user uploaded files. The Galaxy module utilizes this system to ensure that end users do not overload the server with unused older files. Site administrators must set quotas and expiration days based on expected demand and the size of the storage available.

To help support adoption by site administrators, Table 2 provides a listing of useful resources available and related to the Tripal Galaxy module. These include URLs for the online documentation and source code and a set of predefined Galaxy workflows developed by the Hardwood Genomics team. These workflows are available for anyone to download and use on their site. The list also contains URLs to resources for ancillary tools such as Aurora Tools, Blend4php, Tripal documentation and Galaxy documentation. Site developers who have questions, encounter bugs or want to request new features can use the Tripal Galaxy source code repository issue queue to submit these items.

### Security

A Tripal site can communicate with any number of remote Galaxy instances. To do this, the site administrator must add the API key of the Galaxy user account for which the workflows will be executed. This API key enables anyone with it to interact with the remote Galaxy server on behalf of the user. Therefore, to secure this API key, the Tripal Galaxy module provides a security role, compatible with Drupal, that allows a site administrator to limit the set of users that have access to view the key. Ideally, this should only be site administrators. End users will not see the API key and are restricted from viewing administrative pages where the API key is visible. Workflow results are protected such that only the site administrator and the user who submitted the job request can view workflow results.

In general, Tripal depends on Drupal to provide overall site security from attacks such as SQL injection and others. Drupal developers post security updates when new vulnerabilities are found. Also, Drupal coding standards provide mechanisms that, if followed, ensure that Drupal sites are secure. Both the Tripal core package and the Tripal Galaxy module follow these coding standards. Therefore, any Tripal-based site should remain secure if the site administrator maintains and patches the software when security updates are released.

However, any site, no matter how secure, may be compromised, potentially exposing the API key and other sensitive data. Moreover, it is recommended that the Galaxy account provided to the Tripal Galaxy module is a ‘service’ account, pre-created specifically for Tripal to submit jobs, and should never be the API key of an actual user. The Tripal Galaxy module automatically cleans old results, so if a site is compromised results from the most recent job submissions could be accessed. We recommend sites inform users of such vulnerabilities.

Finally, neither Tripal nor Galaxy can ensure security for sensitive data, such as human identifying information or data that might fall, for example, within the regulations such as the Health Insurance Portability and Accountability Act (HIPAA) of the USA. Tripal sites that provide analytical tools to end users should discourage submission of such data to their workflows.

## Challenges

While the Tripal Galaxy module does provide important functionality to support execution of scientific workflows from a Tripal website, several challenges remain. The most significant is that of scale related to demands on hardware. Galaxy can scale well when enough resources are available, but with large data, CPU, RAM and storage can be overrun if site administrators do not set limitations. Determining these limitations can be difficult. To help with storage challenges, Galaxy workflow developers who intend to share their workflows for repeated use should take care to build in cleanup steps to remove unwanted intermediate files, and perhaps input files. Additionally, network bandwidth limitations may create bottlenecks, when a user desires to analyze large amounts of data, such as hundreds of RNA-seq datasets, for example. A Tripal-site perhaps would not be able to support such an extremely large dataset. In cases where a Tripal site could support larger data, the network bandwidth required may be prohibitive.

Access to a responsive Galaxy instance with sufficient resources can also be problematic. Site developers can install their own Galaxy instances provided they have enough computational resources. They may also take advantage of public Galaxy services such as the UseGalaxy.org service. However, public services may not perform well when very busy, may not have all the tools needed or may not want to support a community of users behind a single Tripal-site user account.

## Conclusions

The Tripal Galaxy module strives to meet the need for Tripal-based sites that wish to provide complex analytical workflows for larger data sets. The module provides cyberinfrastructure for scientific workflow execution to perhaps less-funded communities that use the Tripal framework. It helps support sustainability of these sites by adding functionality their site developers need without having to create it themselves. It can also help improve the quality of data submissions for a site, support technical and analytical training and serve as a toolkit to power online research web applications.
